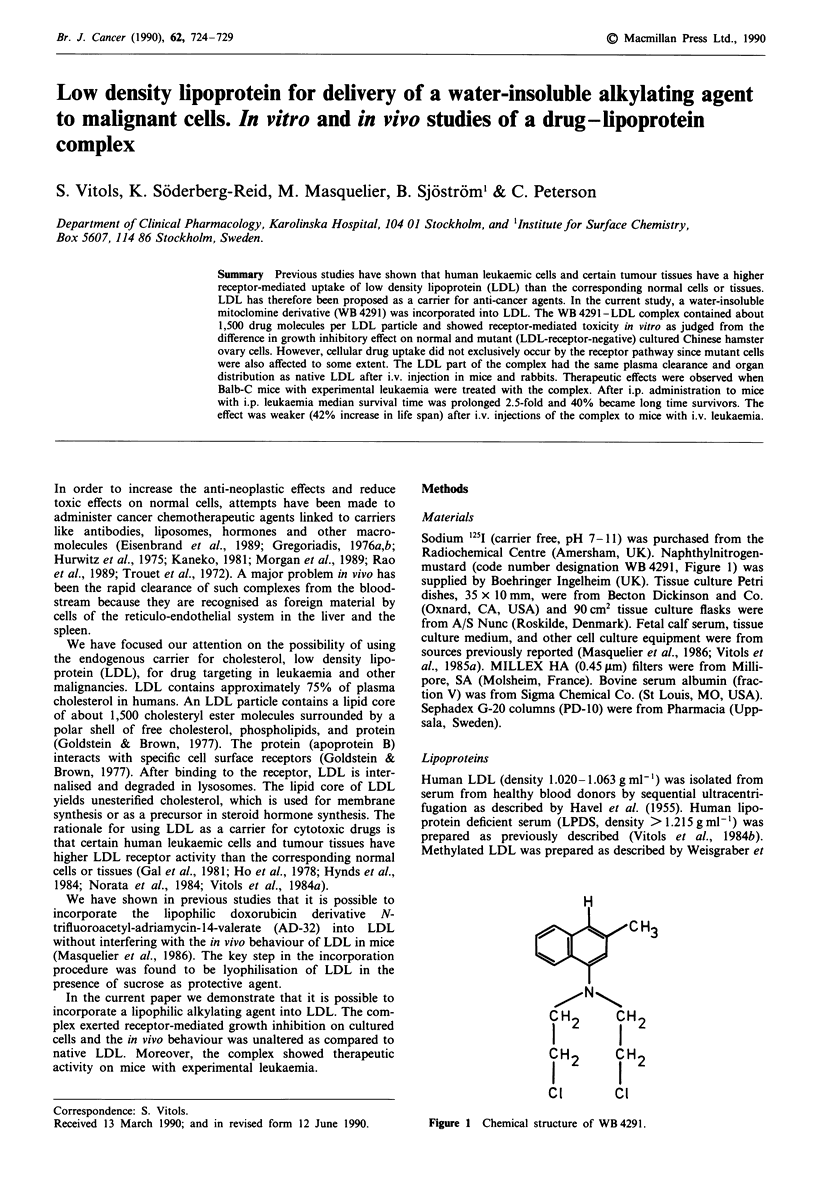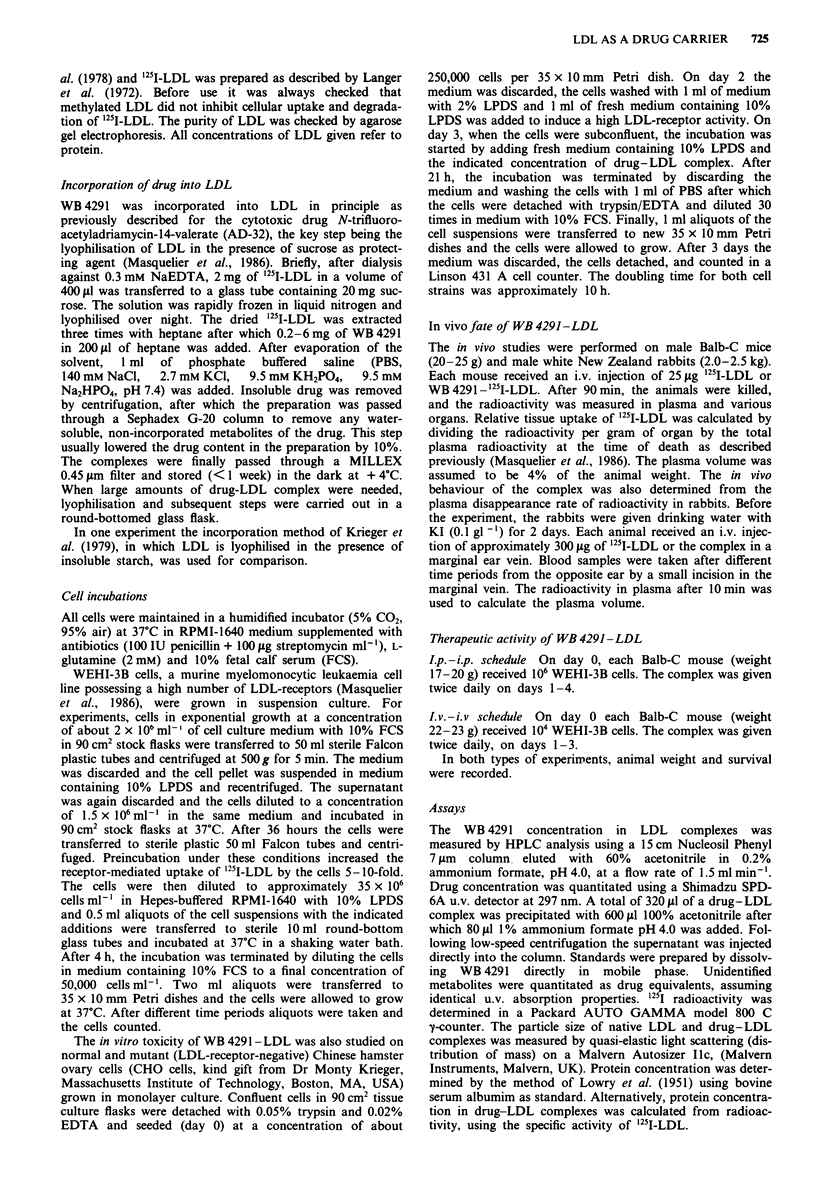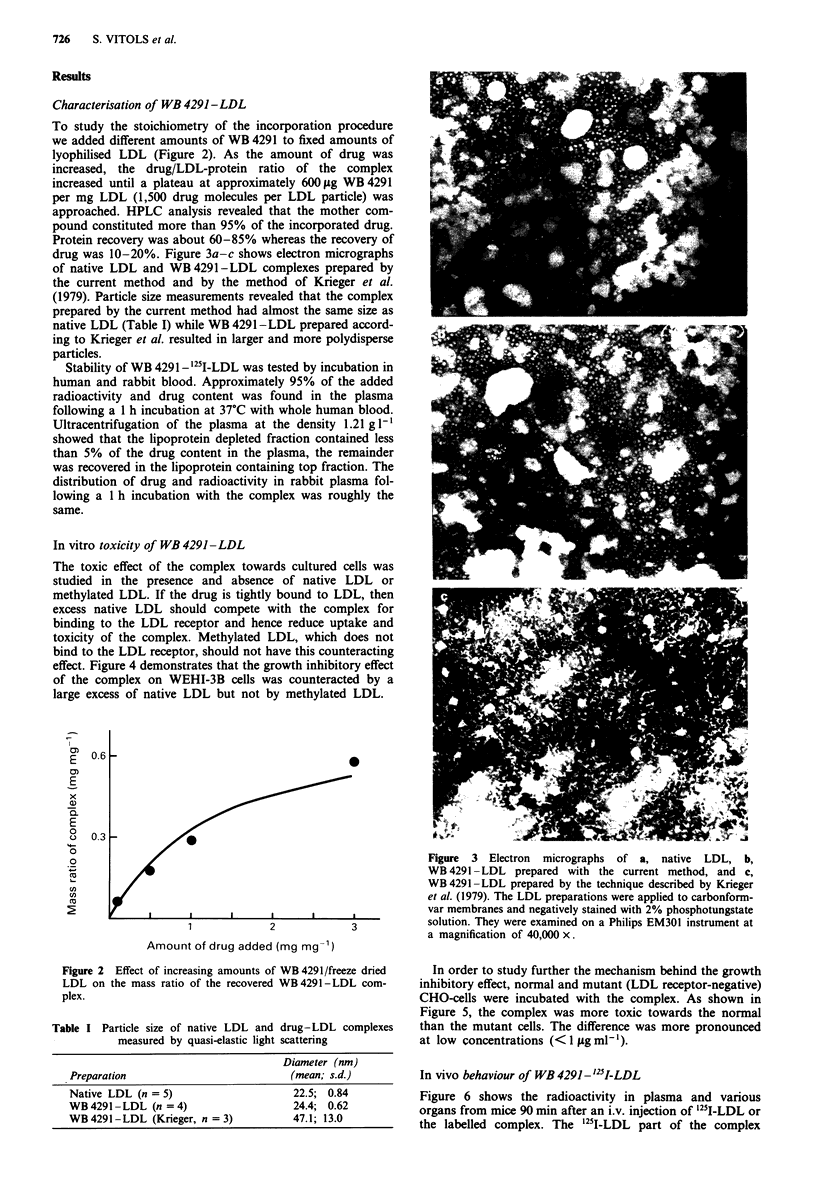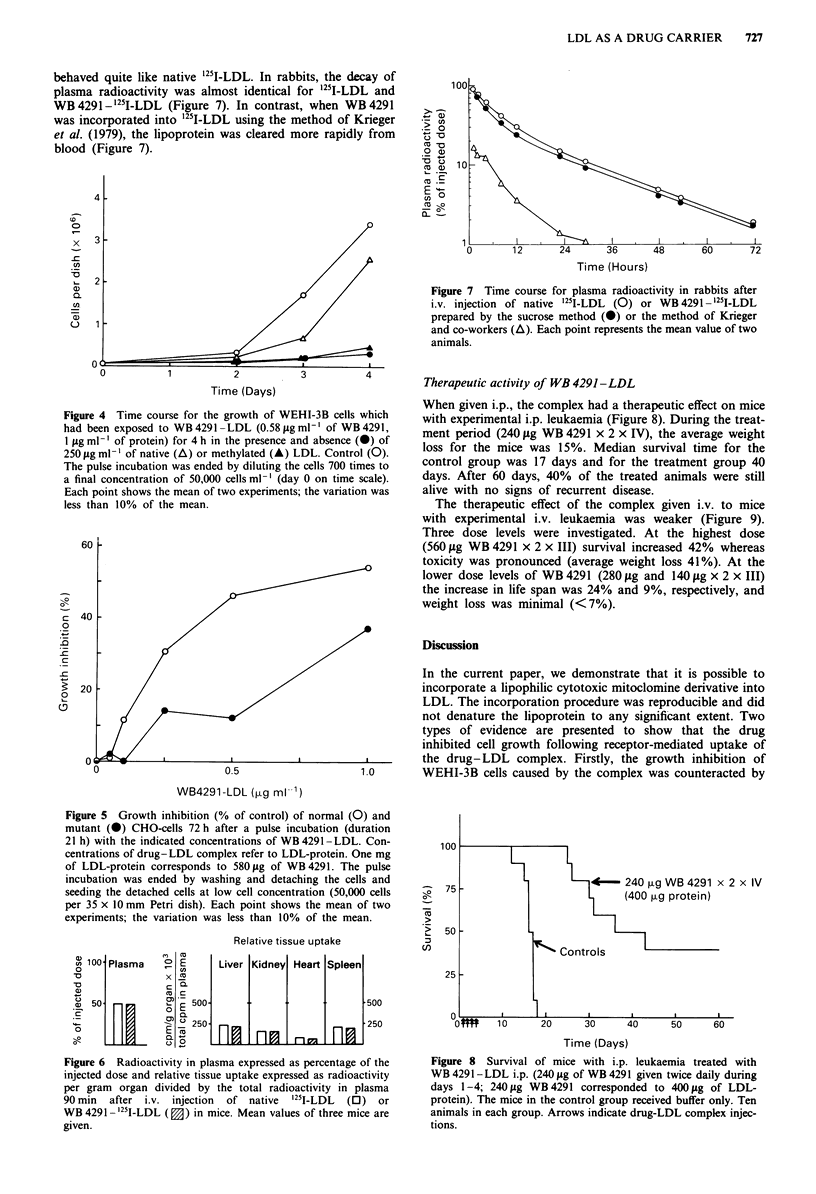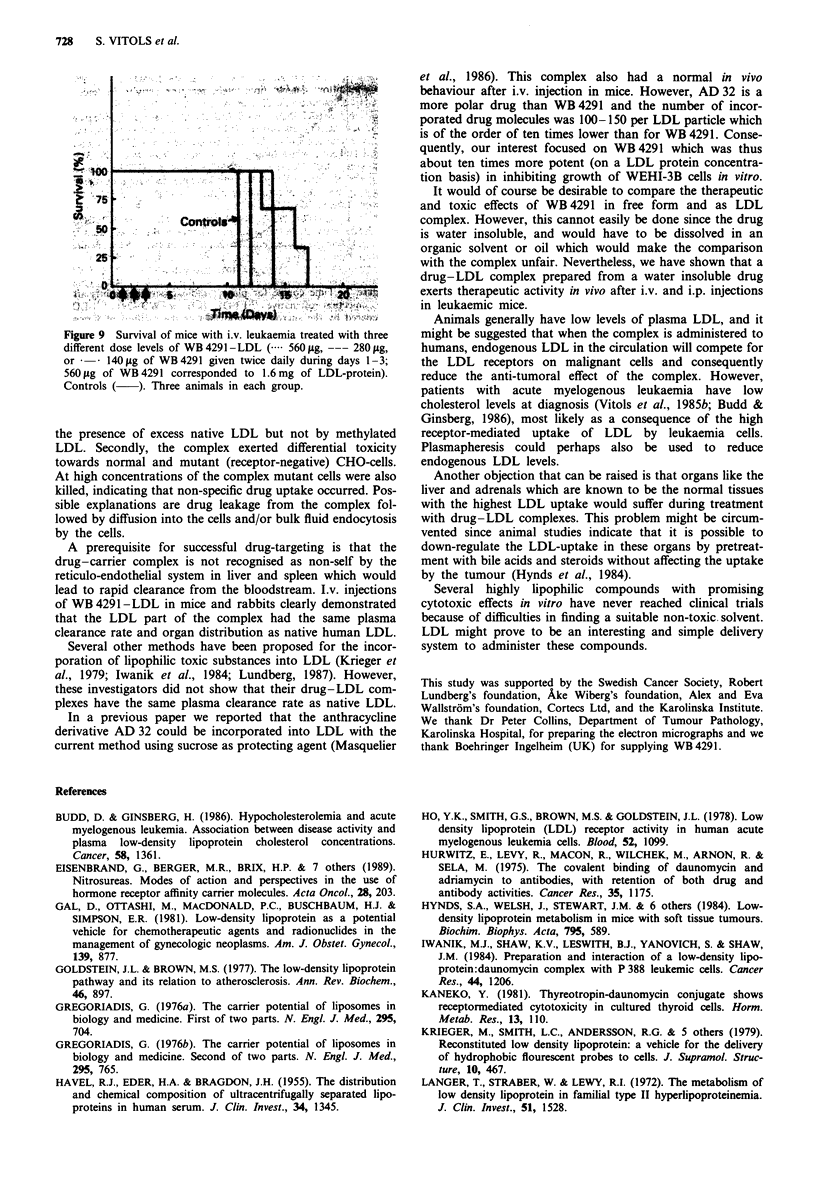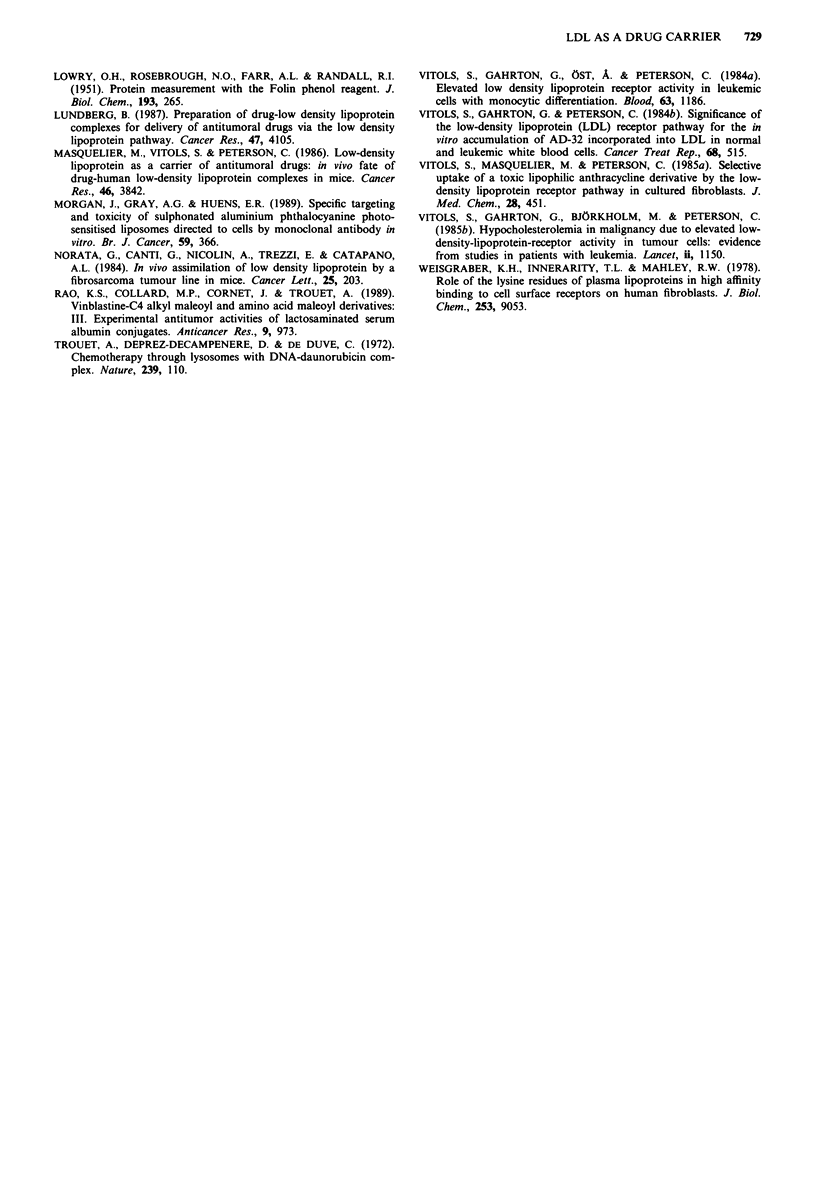# Low density lipoprotein for delivery of a water-insoluble alkylating agent to malignant cells. In vitro and in vivo studies of a drug-lipoprotein complex.

**DOI:** 10.1038/bjc.1990.367

**Published:** 1990-11

**Authors:** S. Vitols, K. Söderberg-Reid, M. Masquelier, B. Sjöström, C. Peterson

**Affiliations:** Department of Clinical Pharmacology, Karolinska Hospital, Stockholm, Sweden.

## Abstract

**Images:**


Br. J. Cancer (1990), 62, 724-729 © Macmillan Press Ltd., 1990

### Low density lipoprotein for delivery of a water-insoluble alkylating agent to malignant cells. *In vitro* and *in vivo* studies of a drug-lipoprotein complex

S. Vitols, K. Söderberg-Reid, M. Masquelier, B. Sjöström[1] & C. Peterson

*Department of Clinical Pharmacology, Karolinska Hospital, 104 01 Stockholm, and [1]Institute for Surface Chemistry, Box 5607, 114 86 Stockholm, Sweden.*

**Summary** Previous studies have shown that human leukaemic cells and certain tumour tissues have a higher receptor-mediated uptake of low density lipoprotein (LDL) than the corresponding normal cells or tissues. LDL has therefore been proposed as a carrier for anti-cancer agents. In the current study, a water-insoluble mitoclomine derivative (WB 4291) was incorporated into LDL. The WB 4291-LDL complex contained about 1,500 drug molecules per LDL particle and showed receptor-mediated toxicity *in vitro* as judged from the difference in growth inhibitory effect on normal and mutant (LDL-receptor-negative) cultured Chinese hamster ovary cells. However, cellular drug uptake did not exclusively occur by the receptor pathway since mutant cells were also affected to some extent. The LDL part of the complex had the same plasma clearance and organ distribution as native LDL after i.v. injection in mice and rabbits. Therapeutic effects were observed when Balb-C mice with experimental leukaemia were treated with the complex. After i.p. administration to mice with i.p. leukaemia median survival time was prolonged 2.5-fold and 40% became long time survivors. The effect was weaker (42% increase in life span) after i.v. injections of the complex to mice with i.v. leukaemia.

In order to increase the anti-neoplastic effects and reduce toxic effects on normal cells, attempts have been made to administer cancer chemotherapeutic agents linked to carriers like antibodies, liposomes, hormones and other macromolecules (Eisenbrand *et al.*, 1989; Gregoriadis, 1976*a*,*b*; Hurwitz *et al.*, 1975; Kaneko, 1981; Morgan *et al.*, 1989; Rao *et al.*, 1989; Trouet *et al.*, 1972). A major problem *in vivo* has been the rapid clearance of such complexes from the bloodstream because they are recognised as foreign material by cells of the reticulo-endothelial system in the liver and the spleen.

We have focused our attention on the possibility of using the endogenous carrier for cholesterol, low density lipoprotein (LDL), for drug targeting in leukaemia and other malignancies. LDL contains approximately 75% of plasma cholesterol in humans. An LDL particle contains a lipid core of about 1,500 cholesteryl ester molecules surrounded by a polar shell of free cholesterol, phospholipids, and protein (Goldstein & Brown, 1977). The protein (apoprotein B) interacts with specific cell surface receptors (Goldstein & Brown, 1977). After binding to the receptor, LDL is internalised and degraded in lysosomes. The lipid core of LDL yields unesterified cholesterol, which is used for membrane synthesis or as a precursor in steroid hormone synthesis. The rationale for using LDL as a carrier for cytotoxic drugs is that certain human leukaemic cells and tumour tissues have higher LDL receptor activity than the corresponding normal cells or tissues (Gal *et al.*, 1981; Ho *et al.*, 1978; Hynds *et al.*, 1984; Norata *et al.*, 1984; Vitols *et al.*, 1984*a*).

We have shown in previous studies that it is possible to incorporate the lipophilic doxorubicin derivative *N*-trifluoroacetyl-adriamycin-14-valerate (AD-32) into LDL without interfering with the *in vivo* behaviour of LDL in mice (Masquelier *et al.*, 1986). The key step in the incorporation procedure was found to be lyophilisation of LDL in the presence of sucrose as protective agent.

In the current paper we demonstrate that it is possible to incorporate a lipophilic alkylating agent into LDL. The complex exerted receptor-mediated growth inhibition on cultured cells and the *in vivo* behaviour was unaltered as compared to native LDL. Moreover, the complex showed therapeutic activity on mice with experimental leukaemia.

Correspondence: S. Vitols.
Received 13 March 1990; and in revised form 12 June 1990.

### Methods

### *Materials*

Sodium $^{125}$I (carrier free, pH 7-11) was purchased from the Radiochemical Centre (Amersham, UK). Naphthylnitrogen-mustard (code number designation WB 4291, Figure 1) was supplied by Boehringer Ingelheim (UK). Tissue culture Petri dishes, 35 × 10 mm, were from Becton Dickinson and Co. (Oxnard, CA, USA) and 90 cm$^2$ tissue culture flasks were from A/S Nunc (Roskilde, Denmark). Fetal calf serum, tissue culture medium, and other cell culture equipment were from sources previously reported (Masquelier *et al.*, 1986; Vitols *et al.*, 1985*a*). MILLEX HA (0.45 µm) filters were from Millipore, SA (Molsheim, France). Bovine serum albumin (fraction V) was from Sigma Chemical Co. (St Louis, MO, USA). Sephadex G-20 columns (PD-10) were from Pharmacia (Uppsala, Sweden).

### *Lipoproteins*

Human LDL (density 1.020-1.063 g ml$^{-1}$) was isolated from serum from healthy blood donors by sequential ultracentrifugation as described by Havel *et al.* (1955). Human lipoprotein deficient serum (LPDS, density > 1.215 g ml$^{-1}$) was prepared as previously described (Vitols *et al.*, 1984*b*). Methylated LDL was prepared as described by Weisgraber *et*

**Figure 1** Chemical structure of WB 4291.

*al.* (1978) and $^{125}$I-LDL was prepared as described by Langer *et al.* (1972). Before use it was always checked that methylated LDL did not inhibit cellular uptake and degradation of $^{125}$I-LDL. The purity of LDL was checked by agarose gel electrophoresis. All concentrations of LDL given refer to protein.

### *Incorporation of drug into LDL*

WB 4291 was incorporated into LDL in principle as previously described for the cytotoxic drug *N*-trifluoroacetyladriamycin-14-valerate (AD-32), the key step being the lyophilisation of LDL in the presence of sucrose as protecting agent (Masquelier *et al.*, 1986). Briefly, after dialysis against 0.3 mM NaEDTA, 2 mg of $^{125}$I-LDL in a volume of 400 μl was transferred to a glass tube containing 20 mg sucrose. The solution was rapidly frozen in liquid nitrogen and lyophilised over night. The dried $^{125}$I-LDL was extracted three times with heptane after which 0.2–6 mg of WB 4291 in 200 μl of heptane was added. After evaporation of the solvent, 1 ml of phosphate buffered saline (PBS, 140 mM NaCl, 2.7 mM KCl, 9.5 mM $KH_2PO_4$, 9.5 mM $Na_2HPO_4$, pH 7.4) was added. Insoluble drug was removed by centrifugation, after which the preparation was passed through a Sephadex G-20 column to remove any water-soluble, non-incorporated metabolites of the drug. This step usually lowered the drug content in the preparation by 10%. The complexes were finally passed through a MILLEX 0.45 μm filter and stored (< 1 week) in the dark at + 4°C. When large amounts of drug-LDL complex were needed, lyophilisation and subsequent steps were carried out in a round-bottomed glass flask.

In one experiment the incorporation method of Krieger *et al.* (1979), in which LDL is lyophilised in the presence of insoluble starch, was used for comparison.

### *Cell incubations*

All cells were maintained in a humidified incubator (5% $CO_2$, 95% air) at 37°C in RPMI-1640 medium supplemented with antibiotics (100 IU penicillin + 100 μg streptomycin $ml^{-1}$), L-glutamine (2 mM) and 10% fetal calf serum (FCS).

WEHI-3B cells, a murine myelomonocytic leukaemia cell line possessing a high number of LDL-receptors (Masquelier *et al.*, 1986), were grown in suspension culture. For experiments, cells in exponential growth at a concentration of about $2 \times 10^6$ $ml^{-1}$ of cell culture medium with 10% FCS in 90 $cm^2$ stock flasks were transferred to 50 ml sterile Falcon plastic tubes and centrifuged at 500 *g* for 5 min. The medium was discarded and the cell pellet was suspended in medium containing 10% LPDS and recentrifuged. The supernatant was again discarded and the cells diluted to a concentration of $1.5 \times 10^6$ $ml^{-1}$ in the same medium and incubated in 90 $cm^2$ stock flasks at 37°C. After 36 hours the cells were transferred to sterile plastic 50 ml Falcon tubes and centrifuged. Preincubation under these conditions increased the receptor-mediated uptake of $^{125}$I-LDL by the cells 5–10-fold. The cells were then diluted to approximately $35 \times 10^6$ cells $ml^{-1}$ in Hepes-buffered RPMI-1640 with 10% LPDS and 0.5 ml aliquots of the cell suspensions with the indicated additions were transferred to sterile 10 ml round-bottom glass tubes and incubated at 37°C in a shaking water bath. After 4 h, the incubation was terminated by diluting the cells in medium containing 10% FCS to a final concentration of 50,000 cells $ml^{-1}$. Two ml aliquots were transferred to 35 × 10 mm Petri dishes and the cells were allowed to grow at 37°C. After different time periods aliquots were taken and the cells counted.

The *in vitro* toxicity of WB 4291–LDL was also studied on normal and mutant (LDL-receptor-negative) Chinese hamster ovary cells (CHO cells, kind gift from Dr Monty Krieger, Massachusetts Institute of Technology, Boston, MA, USA) grown in monolayer culture. Confluent cells in 90 $cm^2$ tissue culture flasks were detached with 0.05% trypsin and 0.02% EDTA and seeded (day 0) at a concentration of about 250,000 cells per 35 × 10 mm Petri dish. On day 2 the medium was discarded, the cells washed with 1 ml of medium with 2% LPDS and 1 ml of fresh medium containing 10% LPDS was added to induce a high LDL-receptor activity. On day 3, when the cells were subconfluent, the incubation was started by adding fresh medium containing 10% LPDS and the indicated concentration of drug–LDL complex. After 21 h, the incubation was terminated by discarding the medium and washing the cells with 1 ml of PBS after which the cells were detached with trypsin/EDTA and diluted 30 times in medium with 10% FCS. Finally, 1 ml aliquots of the cell suspensions were transferred to new 35 × 10 mm Petri dishes and the cells were allowed to grow. After 3 days the medium was discarded, the cells detached, and counted in a Linson 431 A cell counter. The doubling time for both cell strains was approximately 10 h.

### In vivo *fate of WB 4291–LDL*

The *in vivo* studies were performed on male Balb-C mice (20–25 g) and male white New Zealand rabbits (2.0–2.5 kg). Each mouse received an i.v. injection of 25 μg $^{125}$I-LDL or WB 4291–$^{125}$I-LDL. After 90 min, the animals were killed, and the radioactivity was measured in plasma and various organs. Relative tissue uptake of $^{125}$I-LDL was calculated by dividing the radioactivity per gram of organ by the total plasma radioactivity at the time of death as described previously (Masquelier *et al.*, 1986). The plasma volume was assumed to be 4% of the animal weight. The *in vivo* behaviour of the complex was also determined from the plasma disappearance rate of radioactivity in rabbits. Before the experiment, the rabbits were given drinking water with KI (0.1 g$l^{-1}$) for 2 days. Each animal received an i.v. injection of approximately 300 μg of $^{125}$I-LDL or the complex in a marginal ear vein. Blood samples were taken after different time periods from the opposite ear by a small incision in the marginal vein. The radioactivity in plasma after 10 min was used to calculate the plasma volume.

### *Therapeutic activity of WB 4291–LDL*

*I.p.–i.p. schedule* On day 0, each Balb-C mouse (weight 17–20 g) received $10^6$ WEHI-3B cells. The complex was given twice daily on days 1–4.

*I.v.–i.v schedule* On day 0 each Balb-C mouse (weight 22–23 g) received $10^4$ WEHI-3B cells. The complex was given twice daily, on days 1–3.

In both types of experiments, animal weight and survival were recorded.

### *Assays*

The WB 4291 concentration in LDL complexes was measured by HPLC analysis using a 15 cm Nucleosil Phenyl 7 μm column eluted with 60% acetonitrile in 0.2% ammonium formate, pH 4.0, at a flow rate of 1.5 ml $min^{-1}$. Drug concentration was quantitated using a Shimadzu SPD-6A u.v. detector at 297 nm. A total of 320 μl of a drug–LDL complex was precipitated with 600 μl 100% acetonitrile after which 80 μl 1% ammonium formate pH 4.0 was added. Following low-speed centrifugation the supernatant was injected directly into the column. Standards were prepared by dissolving WB 4291 directly in mobile phase. Unidentified metabolites were quantitated as drug equivalents, assuming identical u.v. absorption properties. $^{125}$I radioactivity was determined in a Packard AUTO GAMMA model 800 C γ-counter. The particle size of native LDL and drug–LDL complexes was measured by quasi-elastic light scattering (distribution of mass) on a Malvern Autosizer IIc, (Malvern Instruments, Malvern, UK). Protein concentration was determined by the method of Lowry *et al.* (1951) using bovine serum albumim as standard. Alternatively, protein concentration in drug–LDL complexes was calculated from radioactivity, using the specific activity of $^{125}$I-LDL.

### Results

### *Characterisation of WB 4291–LDL*

To study the stoichiometry of the incorporation procedure we added different amounts of WB 4291 to fixed amounts of lyophilised LDL (Figure 2). As the amount of drug was increased, the drug/LDL-protein ratio of the complex increased until a plateau at approximately 600 µg WB 4291 per mg LDL (1,500 drug molecules per LDL particle) was approached. HPLC analysis revealed that the mother compound constituted more than 95% of the incorporated drug. Protein recovery was about 60–85% whereas the recovery of drug was 10–20%. Figure 3*a–c* shows electron micrographs of native LDL and WB 4291–LDL complexes prepared by the current method and by the method of Krieger *et al.* (1979). Particle size measurements revealed that the complex prepared by the current method had almost the same size as native LDL (Table I) while WB 4291–LDL prepared according to Krieger *et al.* resulted in larger and more polydisperse particles.

Stability of WB 4291–$^{125}$I-LDL was tested by incubation in human and rabbit blood. Approximately 95% of the added radioactivity and drug content was found in the plasma following a 1 h incubation at 37°C with whole human blood. Ultracentrifugation of the plasma at the density 1.21 g l$^{-1}$ showed that the lipoprotein depleted fraction contained less than 5% of the drug content in the plasma, the remainder was recovered in the lipoprotein containing top fraction. The distribution of drug and radioactivity in rabbit plasma following a 1 h incubation with the complex was roughly the same.

### In vitro *toxicity of WB 4291–LDL*

The toxic effect of the complex towards cultured cells was studied in the presence and absence of native LDL or methylated LDL. If the drug is tightly bound to LDL, then excess native LDL should compete with the complex for binding to the LDL receptor and hence reduce uptake and toxicity of the complex. Methylated LDL, which does not bind to the LDL receptor, should not have this counteracting effect. Figure 4 demonstrates that the growth inhibitory effect of the complex on WEHI-3B cells was counteracted by a large excess of native LDL but not by methylated LDL.

**Figure 2** Effect of increasing amounts of WB 4291/freeze dried LDL on the mass ratio of the recovered WB 4291–LDL complex.

**Table I** Particle size of native LDL and drug–LDL complexes measured by quasi-elastic light scattering

| *Preparation* | *Diameter (nm) (mean; s.d.)* |
|---|---|
| Native LDL ($n$ = 5) | 22.5; 0.84 |
| WB 4291–LDL ($n$ = 4) | 24.4; 0.62 |
| WB 4291–LDL (Krieger, $n$ = 3) | 47.1; 13.0 |

**Figure 3** Electron micrographs of **a**, native LDL, **b**, WB 4291–LDL prepared with the current method, and **c**, WB 4291–LDL prepared by the technique described by Krieger *et al.* (1979). The LDL preparations were applied to carbonformvar membranes and negatively stained with 2% phosphotungstate solution. They were examined on a Philips EM301 instrument at a magnification of 40,000 ×.

In order to study further the mechanism behind the growth inhibitory effect, normal and mutant (LDL receptor-negative) CHO-cells were incubated with the complex. As shown in Figure 5, the complex was more toxic towards the normal than the mutant cells. The difference was more pronounced at low concentrations ($<1$ µg ml$^{-1}$).

### In vivo *behaviour of WB 4291–$^{125}$I-LDL*

Figure 6 shows the radioactivity in plasma and various organs from mice 90 min after an i.v. injection of $^{125}$I-LDL or the labelled complex. The $^{125}$I-LDL part of the complex behaved quite like native $^{125}$I-LDL. In rabbits, the decay of plasma radioactivity was almost identical for $^{125}$I-LDL and WB 4291-$^{125}$I-LDL (Figure 7). In contrast, when WB 4291 was incorporated into $^{125}$I-LDL using the method of Krieger *et al.* (1979), the lipoprotein was cleared more rapidly from blood (Figure 7).

**Figure 4** Time course for the growth of WEHI-3B cells which had been exposed to WB 4291-LDL (0.58 µg ml$^{-1}$ of WB 4291, 1 µg ml$^{-1}$ of protein) for 4 h in the presence and absence (●) of 250 µg ml$^{-1}$ of native (△) or methylated (▲) LDL. Control (○). The pulse incubation was ended by diluting the cells 700 times to a final concentration of 50,000 cells ml$^{-1}$ (day 0 on time scale). Each point shows the mean of two experiments; the variation was less than 10% of the mean.

**Figure 5** Growth inhibition (% of control) of normal (○) and mutant (●) CHO-cells 72 h after a pulse incubation (duration 21 h) with the indicated concentrations of WB 4291-LDL. Concentrations of drug-LDL complex refer to LDL-protein. One mg of LDL-protein corresponds to 580 µg of WB 4291. The pulse incubation was ended by washing and detaching the cells and seeding the detached cells at low cell concentration (50,000 cells per 35 × 10 mm Petri dish). Each point shows the mean of two experiments; the variation was less than 10% of the mean.

**Figure 6** Radioactivity in plasma expressed as percentage of the injected dose and relative tissue uptake expressed as radioactivity per gram organ divided by the total radioactivity in plasma 90 min after i.v. injection of native $^{125}$I-LDL (□) or WB 4291-$^{125}$I-LDL (▨) in mice. Mean values of three mice are given.

**Figure 7** Time course for plasma radioactivity in rabbits after i.v. injection of native $^{125}$I-LDL (○) or WB 4291-$^{125}$I-LDL prepared by the sucrose method (●) or the method of Krieger and co-workers (△). Each point represents the mean value of two animals.

*Therapeutic activity of WB 4291-LDL*

When given i.p., the complex had a therapeutic effect on mice with experimental i.p. leukaemia (Figure 8). During the treatment period (240 µg WB 4291 × 2 × IV), the average weight loss for the mice was 15%. Median survival time for the control group was 17 days and for the treatment group 40 days. After 60 days, 40% of the treated animals were still alive with no signs of recurrent disease.

The therapeutic effect of the complex given i.v. to mice with experimental i.v. leukaemia was weaker (Figure 9). Three dose levels were investigated. At the highest dose (560 µg WB 4291 × 2 × III) survival increased 42% whereas toxicity was pronounced (average weight loss 41%). At the lower dose levels of WB 4291 (280 µg and 140 µg × 2 × III) the increase in life span was 24% and 9%, respectively, and weight loss was minimal (<7%).

### Discussion

In the current paper, we demonstrate that it is possible to incorporate a lipophilic cytotoxic mitoclomine derivative into LDL. The incorporation procedure was reproducible and did not denature the lipoprotein to any significant extent. Two types of evidence are presented to show that the drug inhibited cell growth following receptor-mediated uptake of the drug-LDL complex. Firstly, the growth inhibition of WEHI-3B cells caused by the complex was counteracted by

**Figure 8** Survival of mice with i.p. leukaemia treated with WB 4291-LDL i.p. (240 µg of WB 4291 given twice daily during days 1-4; 240 µg WB 4291 corresponded to 400 µg of LDL-protein). The mice in the control group received buffer only. Ten animals in each group. Arrows indicate drug-LDL complex injections.

**Figure 9** Survival of mice with i.v. leukaemia treated with three different dose levels of WB 4291-LDL (···· 560 μg, --- 280 μg, or ·—· 140 μg of WB 4291 given twice daily during days 1-3; 560 μg of WB 4291 corresponded to 1.6 mg of LDL-protein). Controls (——). Three animals in each group.

the presence of excess native LDL but not by methylated LDL. Secondly, the complex exerted differential toxicity towards normal and mutant (receptor-negative) CHO-cells. At high concentrations of the complex mutant cells were also killed, indicating that non-specific drug uptake occurred. Possible explanations are drug leakage from the complex followed by diffusion into the cells and/or bulk fluid endocytosis by the cells.

A prerequisite for successful drug-targeting is that the drug-carrier complex is not recognised as non-self by the reticulo-endothelial system in liver and spleen which would lead to rapid clearance from the bloodstream. I.v. injections of WB 4291-LDL in mice and rabbits clearly demonstrated that the LDL part of the complex had the same plasma clearance rate and organ distribution as native human LDL.

Several other methods have been proposed for the incorporation of lipophilic toxic substances into LDL (Krieger *et al.*, 1979; Iwanik *et al.*, 1984; Lundberg, 1987). However, these investigators did not show that their drug-LDL complexes have the same plasma clearance rate as native LDL.

In a previous paper we reported that the anthracycline derivative AD 32 could be incorporated into LDL with the current method using sucrose as protecting agent (Masquelier *et al.*, 1986). This complex also had a normal *in vivo* behaviour after i.v. injection in mice. However, AD 32 is a more polar drug than WB 4291 and the number of incorporated drug molecules was 100-150 per LDL particle which is of the order of ten times lower than for WB 4291. Consequently, our interest focused on WB 4291 which was thus about ten times more potent (on a LDL protein concentration basis) in inhibiting growth of WEHI-3B cells *in vitro*.

It would of course be desirable to compare the therapeutic and toxic effects of WB 4291 in free form and as LDL complex. However, this cannot easily be done since the drug is water insoluble, and would have to be dissolved in an organic solvent or oil which would make the comparison with the complex unfair. Nevertheless, we have shown that a drug-LDL complex prepared from a water insoluble drug exerts therapeutic activity *in vivo* after i.v. and i.p. injections in leukaemic mice.

Animals generally have low levels of plasma LDL, and it might be suggested that when the complex is administered to humans, endogenous LDL in the circulation will compete for the LDL receptors on malignant cells and consequently reduce the anti-tumoral effect of the complex. However, patients with acute myelogenous leukaemia have low cholesterol levels at diagnosis (Vitols *et al.*, 1985*b*; Budd & Ginsberg, 1986), most likely as a consequence of the high receptor-mediated uptake of LDL by leukaemia cells. Plasmapheresis could perhaps also be used to reduce endogenous LDL levels.

Another objection that can be raised is that organs like the liver and adrenals which are known to be the normal tissues with the highest LDL uptake would suffer during treatment with drug-LDL complexes. This problem might be circumvented since animal studies indicate that it is possible to down-regulate the LDL-uptake in these organs by pretreatment with bile acids and steroids without affecting the uptake by the tumour (Hynds *et al.*, 1984).

Several highly lipophilic compounds with promising cytotoxic effects *in vitro* have never reached clinical trials because of difficulties in finding a suitable non-toxic solvent. LDL might prove to be an interesting and simple delivery system to administer these compounds.

This study was supported by the Swedish Cancer Society, Robert Lundberg's foundation, Åke Wiberg's foundation, Alex and Eva Wallström's foundation, Cortecs Ltd, and the Karolinska Institute. We thank Dr Peter Collins, Department of Tumour Pathology, Karolinska Hospital, for preparing the electron micrographs and we thank Boehringer Ingelheim (UK) for supplying WB 4291.

### References

BUDD, D. & GINSBERG, H. (1986). Hypocholesterolemia and acute myelogenous leukemia. Association between disease activity and plasma low-density lipoprotein cholesterol concentrations. *Cancer*, **58**, 1361.

EISENBRAND, G., BERGER, M.R., BRIX, H.P. & 7 others (1989). Nitrosureas. Modes of action and perspectives in the use of hormone receptor affinity carrier molecules. *Acta Oncol.*, **28**, 203.

GAL, D., OTTASHI, M., MACDONALD, P.C., BUSCHBAUM, H.J. & SIMPSON, E.R. (1981). Low-density lipoprotein as a potential vehicle for chemotherapeutic agents and radionuclides in the management of gynecologic neoplasms. *Am. J. Obstet. Gynecol.*, **139**, 877.

GOLDSTEIN, J.L. & BROWN, M.S. (1977). The low-density lipoprotein pathway and its relation to atherosclerosis. *Ann. Rev. Biochem.*, **46**, 897.

GREGORIADIS, G. (1976*a*). The carrier potential of liposomes in biology and medicine. First of two parts. *N. Engl. J. Med.*, **295**, 704.

GREGORIADIS, G. (1976*b*). The carrier potential of liposomes in biology and medicine. Second of two parts. *N. Engl. J. Med.*, **295**, 765.

HAVEL, R.J., EDER, H.A. & BRAGDON, J.H. (1955). The distribution and chemical composition of ultracentrifugally separated lipoproteins in human serum. *J. Clin. Invest.*, **34**, 1345.

HO, Y.K., SMITH, G.S., BROWN, M.S. & GOLDSTEIN, J.L. (1978). Low density lipoprotein (LDL) receptor activity in human acute myelogenous leukemia cells. *Blood*, **52**, 1099.

HURWITZ, E., LEVY, R., MACON, R., WILCHEK, M., ARNON, R. & SELA, M. (1975). The covalent binding of daunomycin and adriamycin to antibodies, with retention of both drug and antibody activities. *Cancer Res.*, **35**, 1175.

HYNDS, S.A., WELSH, J., STEWART, J.M. & 6 others (1984). Low-density lipoprotein metabolism in mice with soft tissue tumours. *Biochim. Biophys. Acta*, **795**, 589.

IWANIK, M.J., SHAW, K.V., LESWITH, B.J., YANOVICH, S. & SHAW, J.M. (1984). Preparation and interaction of a low-density lipoprotein:daunomycin complex with P 388 leukemic cells. *Cancer Res.*, **44**, 1206.

KANEKO, Y. (1981). Thyreotropin-daunomycin conjugate shows receptormediated cytotoxicity in cultured thyroid cells. *Horm. Metab. Res.*, **13**, 110.

KRIEGER, M., SMITH, L.C., ANDERSSON, R.G. & 5 others (1979). Reconstituted low density lipoprotein: a vehicle for the delivery of hydrophobic flourescent probes to cells. *J. Supramol. Structure*, **10**, 467.

LANGER, T., STRABER, W. & LEWY, R.I. (1972). The metabolism of low density lipoprotein in familial type II hyperlipoproteinemia. *J. Clin. Invest.*, **51**, 1528.

LOWRY, O.H., ROSEBROUGH, N.O., FARR, A.L. & RANDALL, R.I. (1951). Protein measurement with the Folin phenol reagent. *J. Biol. Chem.*, **193**, 265.

LUNDBERG, B. (1987). Preparation of drug-low density lipoprotein complexes for delivery of antitumoral drugs via the low density lipoprotein pathway. *Cancer Res.*, **47**, 4105.

MASQUELIER, M., VITOLS, S. & PETERSON, C. (1986). Low-density lipoprotein as a carrier of antitumoral drugs: *in vivo* fate of drug-human low-density lipoprotein complexes in mice. *Cancer Res.*, **46**, 3842.

MORGAN, J., GRAY, A.G. & HUENS, E.R. (1989). Specific targeting and toxicity of sulphonated aluminium phthalocyanine photosensitised liposomes directed to cells by monoclonal antibody *in vitro*. *Br. J. Cancer*, **59**, 366.

NORATA, G., CANTI, G., NICOLIN, A., TREZZI, E. & CATAPANO, A.L. (1984). *In vivo* assimilation of low density lipoprotein by a fibrosarcoma tumour line in mice. *Cancer Lett.*, **25**, 203.

RAO, K.S., COLLARD, M.P., CORNET, J. & TROUET, A. (1989). Vinblastine-C4 alkyl maleoyl and amino acid maleoyl derivatives: III. Experimental antitumor activities of lactosaminated serum albumin conjugates. *Anticancer Res.*, **9**, 973.

TROUET, A., DEPREZ-DECAMPENERE, D. & DE DUVE, C. (1972). Chemotherapy through lysosomes with DNA-daunorubicin complex. *Nature*, **239**, 110.

VITOLS, S., GAHRTON, G., ÖST, Å. & PETERSON, C. (1984*a*). Elevated low density lipoprotein receptor activity in leukemic cells with monocytic differentiation. *Blood*, **63**, 1186.

VITOLS, S., GAHRTON, G. & PETERSON, C. (1984*b*). Significance of the low-density lipoprotein (LDL) receptor pathway for the *in vitro* accumulation of AD-32 incorporated into LDL in normal and leukemic white blood cells. *Cancer Treat Rep.*, **68**, 515.

VITOLS, S., MASQUELIER, M. & PETERSON, C. (1985*a*). Selective uptake of a toxic lipophilic anthracycline derivative by the low-density lipoprotein receptor pathway in cultured fibroblasts. *J. Med. Chem.*, **28**, 451.

VITOLS, S., GAHRTON, G., BJÖRKHOLM, M. & PETERSON, C. (1985*b*). Hypocholesterolemia in malignancy due to elevated low-density-lipoprotein-receptor activity in tumour cells: evidence from studies in patients with leukemia. *Lancet*, **ii**, 1150.

WEISGRABER, K.H., INNERARITY, T.L. & MAHLEY, R.W. (1978). Role of the lysine residues of plasma lipoproteins in high affinity binding to cell surface receptors on human fibroblasts. *J. Biol. Chem.*, **253**, 9053.